# Fragmented QRS as a Predictor of Appropriate Implantable Cardioverter-defibrillator Therapy

**DOI:** 10.1016/s0972-6292(16)30710-0

**Published:** 2014-01-01

**Authors:** Sirin Apiyasawat, Dujdao Sahasthas, Tachapong Ngarmukos, Pakorn Chandanamattha, Khanchit Likittanasombat

**Affiliations:** Ramathibodi Hospital, Thailand

**Keywords:** Fragmented QRS, Implantable cardioverter-defibrillator, tachyarrhythmia, electrocardiography

## Abstract

**Background:**

Fragmented QRS (fQRS) has been shown to be a marker of local myocardial conduction abnormalities and a predictor of cardiac events in selected populations. We hypothesized that the presence of a fQRS might predict arrhythmic events in patients who received an implantable cardioverter-defibrillator (ICD), regardless of the indications for implantation.

**Methods and Results:**

A cohort of 107 consecutive patients (mean age, 53 years; 82% male) who underwent an ICD implantation was studied. We defined fQRS, on a routine 12-lead ECG, as the presence of an additional R wave or notching in the nadir of the S wave in 2 consecutive leads corresponding to a major coronary artery territory. In the presence of bundle branch block, more than 2 notches in the R or S waves in 2 consecutive leads were required to characterize fQRS. Patients were followed for 21.3±23 months for appropriate ICD therapy (antitachycardia pacing and/or shock). ICDs were implanted predominantly in patients with ischemic cardiomyopathy (N=45, 42.1%), followed by Brugada syndrome (N=26, 24.3%). fQRS presented in 42 patients (39.3%). During follow-up, patients with fQRS received more appropriate ICD therapy than those without fQRS (45.2% vs. 10.8%, P<0.0001). After adjustment for covariates, fQRS remained an independent predictor for appropriate ICD therapy (hazard ratio=5.32, 95% confidence interval=2.11-13.37, P<0.0001).

**Conclusion:**

The presence of fQRS appeared to be directly associated with appropriate ICD therapy.

## Introduction

Implantable cardioverter-defibrillator (ICD) is by far the most effective therapy for sudden cardiac arrest. According to the current guidelines [1,2], the indications for ICDs are primarily based on a previous history of sudden cardiac arrest or sustained ventricular arrhythmias, certain ECGs features, New York Heart Association (NYHA) class, and left ventricular ejection fraction (LVEF). In spite of using these well-established criteria, a significant number of patients with implanted ICDs have never been treated by their ICDs [3,4]. Several tests have been introduced to further stratify the perfect candidates for ICDs. However, most of these tests provide few incremental benefits over the current criteria [5,6].

Recently, fragmented QRS (fQRS), the presence of fragmentations in the QRS complex on a standard 12-lead ECG, has been shown to be a predictor of mortality and sudden cardiac death [7]. fQRS represents inhomogeneous myocardium and local conduction abnormalities [8,9]. In patients with CAD, fQRS was associated with a prior myocardial scarring [10]. In patients with Brugada syndrome [9], long QT syndrome [11], and non-ischemic cardiomyopathy [12], fQRS was shown to predict arrhythmic events.

In this study, we proposed the potential role of fQRS as a risk stratifier for sudden cardiac arrest. We hypothesized that in patients implanted with ICDs for all indications, the presence of fQRS would be associated with a higher incidence of appropriate ICD therapy.

## Methods

A total of 138 consecutive patients who underwent ICD implantation in our institution were identified. Data from medical records during initial implantation and follow-up were collected. Out of 138 patients, 31 patients were lost during follow-up and were excluded. Therefore, 107 patients remained in the study. All implantations were performed as indicated by standard guidelines. The parameters for ICD detection and therapy were set at the implanters' discretion. The primary endpoint was appropriate ICD therapy, which is defined for therapy for ventricular arrhythmia by either shock or antitachycardia pacing (ATP).

### Fragmented QRS (fQRS)

**Figure 1:**
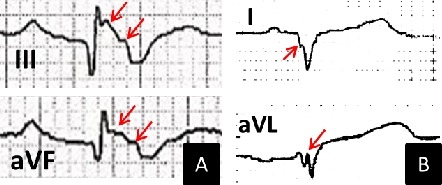
Example of patients with fragmented QRS. A: ECG from a 32-year-old woman with arrhythmogenic right ventricular dysplasia who later received multiple shocks from implantable cardioverter-defibrillator (ICD) due to ventricular fibrillation. B: ECG from a 36-year-old man with non-ischemic cardiomyopathy who later received multiple ICD therapies due to ventricular tachycardia. Fragmentations in QRS complexes are noted with arrows.

We determined the presence of fQRS by using a standard 12-lead ECG (filter range 0.15-100 Hz; AC filter 50 Hz, 25 mm/s, 10 mm/mV) obtained within 6 months of the ICD implantation. According to previous studies [7,9-12], fQRS was defined as the presence of an additional R wave or notching in the nadir of the S wave in 2 consecutive leads corresponding to a major coronary artery territory (Figure 1). In the presence of bundle branch block, more than 2 notches in the R or S waves in 2 consecutive leads were required to characterize fQRS. All ECGs were reviewed by 2 independent readers without knowledge of the patients. There was 99.1% concordance in the interpretation of fQRS.

### Statistical Analysis

The data were expressed as the mean ± SD values for continuous variables and as frequencies for categorical variables. Comparisons of 2 groups were made with Student's t test for continuous variables and Pearson's chi-square test for categorical variables. Survival and event rates were determined using the Kaplan-Meier method and compared between groups with a 2-sample log-rank test. To identify the independent predictors of the primary endpoint, a Cox proportional hazard model was developed. Age, sex, and the variables that had a P value of <0.1 by univariate analysis were entered into the model. The odd ratios (OR) and 95% confidence intervals (CI) were calculated. All statistical analyses were performed with SPSS 11.0 (SPSS Inc., Chicago, IL). Significance was defined as a P value of ≤0.05. The authors had full access to the data and take full responsibility for its integrity. All authors have read and agreed to the manuscript as written.

## Results

The population (N=107, mean age 53) was predominantly male (N=88, 82.2%). More than half of the patients (N=71, 66%) had an ICD implanted for secondary prevention (33 patients with aborted sudden cardiac arrest, 65 patients with documented ventricular arrhythmias, and 35 patients with syncope and positive electrophysiologic study for inducible ventricular arrhythmias). The most common underlying cardiac pathology was ischemic cardiomyopathy (N=45, 42.1%), followed by Brugada syndrome (N=26, 24.3%). fQRS was detected in 42 patients (39.3%). The average number of leads with fQRS was 3.8 leads (range 2-8). Among those with positive electrophysiologic study (N=35), 15 had fQRS (42.9%). Comparing patients with and without fQRS, all had essentially the same demographics, medical history, LVEF, QRS duration, and QT duration (Table 1).

### Events

After a mean follow-up period of 21.2 months, 9 patients (8.4%) died; 8 died due to cardiac cause. The primary endpoint was reached in 26 patients (24.3%); 20 received ICD shock, 11 received ATP, and 5 received both. Of those who received appropriate ICD therapy, 8 had ischemic cardiomyopathy, 7 had Brugada syndrome, 5 had arrhythmogenic right ventricular dysplasia, 4 had non-ischemic cardiomyopathy, and 2 had long QT syndrome. The incidence of appropriate ICD therapy was significantly higher in patients with fQRS than in those without fQRS (45.2% vs. 10.8%, P<0.0001, Table 2). The incidence of fQRS, sensitivity, specificity, positive predictive value (PPV), and negative predictive value (NPV) for appropriate ICD therapy in various subgroups were calculated (Table 3). Except for those with wide QRS, the predictive accuracy were relatively the same across the subgroups.

**Figure 2:**
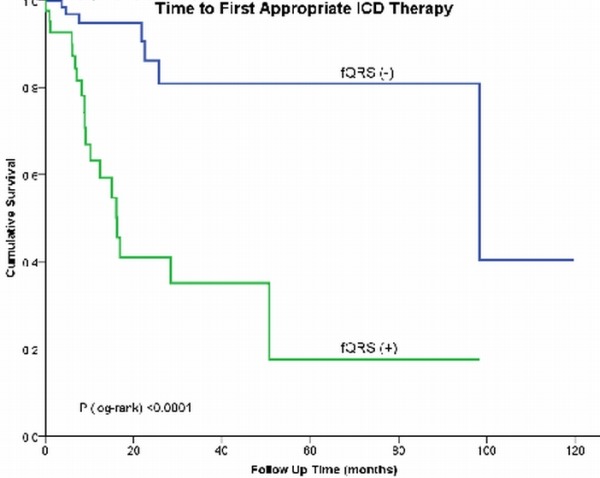
Kaplan-Meier survival analysis for appropriate implantable cardioverter-defibrillator (ICD) therapy in patients with fragmented QRS (fQRS). fQRS (+) = presence of fQRS, fQRS (-) = absence of fQRS.

At the 2-year mark, 94.9% of patients without fQRS were free of appropriate ICD therapy, compared with 66.9% of patients with fQRS (P <0.0001 by log-rank test, Figure 2). After adjusting for other covariates, fQRS was the strongest independent predictor of appropriate ICD therapy (HR 5.318, 95% CI 2.115-13.374, Table 4), followed by Amiodarone use (HR 3.468, 95% CI 1.254-9.595).

## Discussions

In the present study, we showed a strong association between fQRS and ventricular arrhythmic events in patients who had ICD implantation for various indications, as well as for both primary and secondary prevention. Of all patients in the study, 24.3% received appropriate ICD therapy. The incidence increased to 45.2% in patients with fQRS. Independent of the patients' characteristics, the presence of fQRS was associated with more than 5 times the chance of receiving appropriate ICD therapy.

fQRS represents an underlying arrhythmogenic substrate. The distortion and fragmentation of QRS occurs when normally smooth myocardial activation is disrupted [13]. The cause of the disruption may be either structural or functional changes. In a postmortem series, more notches in the QRS complex were detected in an infarcted or an enlarged ventricle than in a normal ventricle [8]. In patients with coronary artery disease, fQRS was shown to be a better marker of prior myocardial infarction than Q waves [7]. In canine models of Brugada syndrome, fQRS resulted from functional delay in epicardial activation, which provided a substrate for ventricular arrhythmia [9]. Various clinical outcome studies have confirmed the potential role of fQRS as a predictor of ventricular arrhythmic events in patients with coronary artery disease, non-ischemic cardiomyopathy, Brugada syndrome, and acquired long QT syndrome [7,9-12]. In a series of Brugada syndrome patients with recurrent ventricular arrhythmias, epicardial mapping showed abnormal low voltage and fractionated late potentials clustering in the anterior part of right ventricular outflow tract. Ablation over this area was shown to prevent recurrent ventricular arrhythmias [14].

Our study population represents a real life population of all-coming patients undergoing ICD implantation. It also represents Asian population where Brugada syndrome is relatively common. The average LVEF was 43%. The predictive accuracy of fQRS in our study was comparable to others (sensitivity and specificity of 73.1% and 71.6%, PPV and NPV of 45.2% and 89.2%, respectively). Morita et al. [9] investigated the utility of fQRS in Brugada syndrome, and fQRS was found in 43% of the patients. The positive and negative predictive values of fQRS for arrhythmic events in that study were 34% and 98.5%, respectively. In another study of ischemic and non-ischemic cardiomyopathy, which involved patients who received ICD for both primary and secondary prevention, the incidence of arrhythmic events in patients with fQRS was 40.5%, which is 7 times higher than that in patients without fQRS [12]. However, in a larger but more specific population, a prospective cohort of 842 patients with LVEF ≤35% who received ICD for primary prevention, fQRS was not associated with a higher risk of arrhythmic mortality [15]. Compared to predictive accuracy of other non-invasive risk stratification tests, fQRS appears to be slightly more positively predictive but less negatively predictive for arrhythmic events than other tests. One meta-analysis of 19 studies on microvolt T-wave alternans estimates the PPV and NPV for arrhythmic events at 19.3% and 97.2%, respectively [16]. Another meta-analysis of 44 studies on signal-averaged ECG and heart rate variability estimated the PPV for life-threatening arrhythmic events in the range of 20-26%, and the NPV in the range of 95-96% [17].

We also showed the predictive accuracy of fQRS in various population. The sensitivity ranged from 66-100%, and the specificity ranged from 68-89%. Of particular interest, fQRS was 100% sensitive in patients with QRS duration of >120 ms. Wide QRS alone has been shown to be a predictor of arrhythmic event [12]. fQRS in wide QRS, however, has been excluded by most of the studies on fQRS. Das et al. analyzed the cardiac events rate in patients with fQRS and included those with wide QRS [7]. Both fQRS and QRS duration were shown to be independent predictors to major cardiac events. But the influence of QRS duration in predictive accuracy of fQRS was not calculated in that trial. This finding might be explained by the electrophysiologic property of amiodarone that delays the action potential duration which may enhance the manifestation of fQRS.

The limitations of our study include the small size of the study population and the bias associated with retrospective study. The utility of fQRS by itself is also limited by the lack of data regarding fQRS in a normal population. It remains unclear whether fQRS would have any prognostic value among patients without cardiac diseases.

## Conclusions

We demonstrated that fQRS, as detected by standard 12-leads ECG, was a prognostic marker for arrhythmic events in patients implanted with ICD, regardless of the indication. Large clinical trials are required to further evaluate the role of fQRS in patient selection for ICD implantation.

## Figures and Tables

**Table 1:**
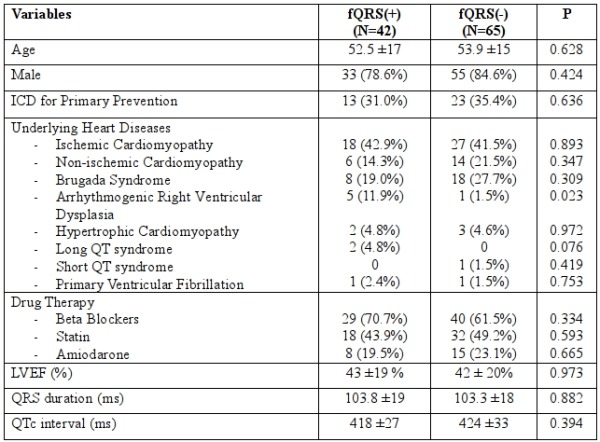
Characteristics of patients with fQRS and without fQRS.

fQRS = fragmented QRS, fQRS (+) = presence of fQRS, fQRS (-) = absence of fQRS, ICD = implantable cardioverter-defibrillator, LVEF = left ventricular ejection fraction

**Table 2:**
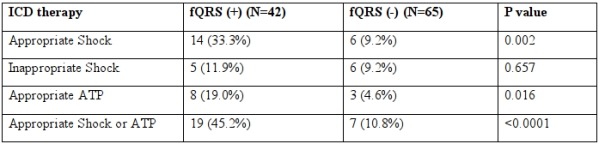
fQRS and incidence of ICD therapy.

fQRS = fragmented QRS, fQRS (+) = presence of fQRS, fQRS (-) = absence of fQRS, ICD = implantable cardioverter-defibrillator, ATP = antitachycardia pacing

**Table 3:**
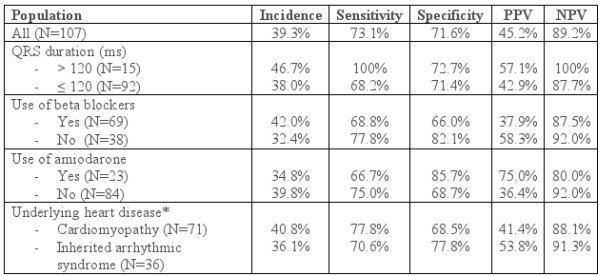
Incidence and predictive accuracy of fQRS for appropriate ICD therapy in various population.

fQRS = fragmented QRS, ICD = implantable cardioverter-defibrillator, PPV = positive predictive value, NPV = negative predictive value, *Cardiomyopathy = ischemic cardiomyopathy, non-ischemic cardiomyopathy, and arrhythmogenic right ventricular dysplasia; inherited arrhythmic syndrome = Brugada syndrome, hypertrophic cardiomyopathy, long QT syndrome, short QT syndrome, and primary ventricular fibrillation.

**Table 4:**
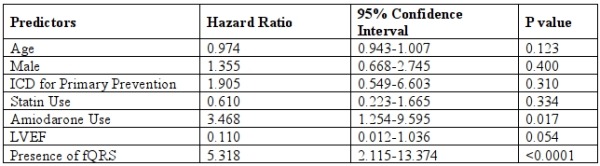
Predictors of appropriate ICD therapy according to a Cox proportional hazard model.

ICD = implantable cardioverter-defibrillator, LVEF = left ventricular ejection fraction, fQRS = fragmented QRS
